# Role of conventional dendritic cells in schistosomiasis-induced pulmonary hypertension

**DOI:** 10.1042/CS20256896

**Published:** 2025-10-14

**Authors:** Claudia Mickael, Rahul Kumar, Dara C. Fonseca Balladares, Kevin Nolan, Michael H. Lee, Biruk Kassa, Thais C.F Menezes, Anthony Lau-Xiao, Ajaypal Sahota, Linda Sanders, Katie Tuscan, Aneta Gandjeva, Kelly M. Cautivo, Ari Molofsky, Brian B. Graham

**Affiliations:** 1 Division of Pulmonary Sciences and Critical Care Medicine / Cardiovascular Research Laboratories, Department of Medicine, University of Colorado Anschutz Medical Campus, Colorado, U.S.A.; 2 Department of Medicine, University of California San Francisco, California, U.S.A.; 3 Lung Biology Center, Zuckerberg San Francisco General Hospital, San Francisco, California, U.S.A.; 4 School of Pharmacy, University of California San Francisco, California, U.S.A.; 5 Division of Respiratory Diseases, Department of Medicine, Federal University of São Paulo, São Paulo, SP, Brazil; 6 Department of Laboratory Medicine, University of California San Francisco, San Francisco, U.S.A.

**Keywords:** dendritic cells, pulmonary hypertension, schistosomiasis

## Abstract

**Background:** Schistosomiasis is a major cause of pulmonary hypertension (PH) worldwide, and CD4 T cells are critical in disease pathogenesis. The role of dendritic cells (DCs) in *Schistosoma*-induced PH (SchPH) is unknown. There are two types of conventional DCs, cDC1 and cDC2, that prototypically activate CD8 and CD4 T cells, respectively. **Methods:** We exposed wildtype, DC reporter, and DC knockout mice to *Schistosoma mansoni* and quantified PH severity by heart catheterization and cell density by flow cytometry. **Results:** Experimental *S. mansoni* exposure increased the density of pulmonary DCs, particularly cDC2s. Deleting both cDC subsets did not significantly modify SchPH disease severity. Deleting only cDC1s caused more severe SchPH, associated with more Th2 CD4 and CD8 T cells. In contrast, deleting only cDC2s reduced SchPH disease severity. **Conclusions:** cDC1s appear to be protective, whereas cDC2s promote disease in SchPH.

## Introduction

Schistosomiasis is a major pulmonary hypertension (PH) etiology worldwide. Type 2 immunity is required for the development of experimental *Schistosoma*-induced PH [1]. More precisely, Th2 CD4 T cells located in the lung and expressing IL-4 and IL-13 specifically are required for *Schistosoma*-PH [2,3].

Dendritic cells (DCs) are professional antigen-presenting cells (APCs) at the interface between innate and adaptive immunity, with the capability of expressing MHC II to activate CD4 T cells. In the lung, there are two major conventional DC (cDC) populations: CD103^+^ (cDC1s) and CD11b^+^ (cDC2s) [4–6], as well as circulating plasmacytoid DCs (pDCs). cDC2s are thought to be the primary cells presenting antigen to CD4^+^ T cells [5,7,8]. In contrast, cDC1s primarily activate CD8 T cells [9]. Other nonprofessional APCs can also express MHC II in certain settings, such as endothelial cells [10].

The types of APCs that contribute to CD4 T cell activation in *Schistosoma*-PH pathogenesis are unknown. The complexity of population-specific APC roles is underscored by the finding that in hepatic *S. mansoni* disease, cDC1-depleted mice (*Batf3^-/-^*) have augmented Th2 inflammation and fibrosis [11]. Importantly, there may be antigen-specific roles: another study reported that *Batf3^-/-^* mice infected with *S. japonicum* had increased Th1 inflammation and unchanged Th2, resulting in less hepatic fibrosis [12]. In PH, *S. mansoni* causes more severe disease than *S. japonicum* [13]. To our knowledge, cDC2s have not been previously investigated in *Schistosoma*-induced lung pathology.

Here, we investigated the potential role of cDC1s and cDC2s in *Schistosoma*-induced PH, using transgenic mice deleted for each population. We hypothesized that DCs contribute to Th2-driven *Schistosoma*-PH. Specifically, we hypothesized that cDC2s would promote Type 2 inflammation, based on the requirement for activated CD4 T cells in this model. In contrast, we hypothesized that cDC1s would be protective against *Schistosoma*-PH, particularly following *S. mansoni* exposure given the data in hepatic disease due to this species [11]. We observed that cDC1-depleted mice (*Batf3* ^-/-^) had worse *S. mansoni*-induced PH. In contrast, depletion of cDC2s (*Cd301b^DTR^*) ameliorated the PH phenotype.

## Methods

### Animals

Wildtype (C57BL6/J; Jackson Labs, Bar Harbor ME, Strain #000664), *Zbtb46^DTR^*, and *Batf3^-/-^* (Jax #013755) mice were purchased. *Cd301b^DTR^* mice were obtained courtesy of Dr. Akiko Iwasaki (Yale University, U.S.A.). The mice were housed at the University of California San Francisco (UCSF) or University of Colorado Anschutz Medical Campus (CUAMC) specific pathogen-free animal facilities.

### Experimental treatments

Mice were experimentally exposed to *Schistosoma mansoni or S. japonicum* eggs following our established protocol [14,15]. *Schistosoma* eggs were obtained from infected mice using standard techniques [16]. Experimental mice were sensitized with 240 eggs/gram intraperitoneally (IP) with *Schistosoma* eggs, followed 14 days later by intravenous (IV) challenge with *Schistosoma* eggs. *Schistosoma*-unexposed mice served as controls. *Zbtb46^DTR^* bone marrow (BM) chimeras were created by lethally irradiating isogenetic wildtype recipients (10 Gy, in two fractions), followed by administering 1–2 × 10^6^ BM cells obtained from the femurs of *Zbtb46^DTR^* (homozygote) donors. The recipients received chow containing trimethoprim sulfa for two weeks, followed by two more weeks of recovery, and then were used for experimentation. To delete both cDC1 and cDC2 populations, *Zbtb46^DTR^* chimera mice were administered IP diphtheria toxin (DT), 40 ng/g and then 8 ng/g every three days later. To delete cDC2s, *CD301b^DTR/+^* mice were administered IP DT, 500 ng every three days. Of note, experiments were performed at Denver elevation (1620 m), other than those with *CD301b^DTR/+^* mice which were performed at sea level.

### PH experimental endpoints

Right ventricular systolic pressure (RVSP) and RV hypertrophy were measured as previously described [14,17]. In brief, mice were sedated with ketamine/xylazine and ventilated via tracheal intubation at 190 breaths/minute with a tidal volume of 200 µl, using 40% inspired oxygen, and maintained on a heating pad during the procedure to avoid hypothermia. The abdominal and thoracic cavities were opened, and a 1Fr pressure-volume catheter (PVR-1035, Millar Instruments, Houston TX) was placed through the right ventricle free wall to transduce the RV pressure. The RVSP measurement was made over an average of 15–20 respiratory cycles to minimize respiratory variability. The Fulton index was assessed by resecting the RV free wall from the left ventricle and septum, weighing them separately, and calculating the ratio. The lung tissue was then harvested and snap-frozen for subsequent protein measurement, or agarose inflated for formalin fixation and paraffin embedding (FFPE).

### Vascular remodeling quantification

Pulmonary vascular remodeling was assessed by immunostaining FFPE tissue for α-smooth muscle actin (Invitrogen 14–9760-82, ThermoFisher, Waltham MA) by published protocol [13]. Images were captured with a Nikon Eclipse 80i microscope, and the vascular media was identified using image processing software (Image-Pro 10, Media Cybernetics, Rockville MD). The average radii of the outer and internal perimeters of the medial layer were quantified, and the fractional medial thickness was calculated as the difference in these radii divided by the external media radius.

### Peri-egg granuloma volume estimation

The optical rotator stereology method [18] was used to estimate peri-e.g.g granuloma volumes surrounding a single e.g.g on images from hematoxylin and eosin stained slides using image processing software (Image-Pro 10).

### Protein quantification

ELISA kits were used for the quantification of IL-4 and IL-13 (R&D Systems, Minneapolis, MN; DY404-05 and DY413-05, respectively, with DY008B ancillary reagents) using whole lung lysates from unexposed and *Schistosoma*-exposed mice, following the manufacturer’s instructions.

### Flow cytometry

All flow cytometry experiments were performed three days after IV challenge to coincide with the peak of inflammation. Mice were initially anesthetized, and a fluorophore-label anti-CD45 antibody was injected retro-orbitally 2 minutes before end of life of mice to distinguish the intravenous from the parenchymal compartments (intravascular cells are IV-CD45+). Flushed lungs were digested with Liberase (Roche-Genentech, South San Francisco, CA) at a concentration of 0.4 mg/ml in RPMI medium. The samples were incubated for 30 minutes at 37°C with 5% CO2. To stop the reaction, 10 mM EDTA in RPMI was added. The tissue was then mechanically disrupted by passing it through a 16-gauge needle five times, followed by an 18-gauge needle five more times. The digested material was filtered through a 100 µm filter, centrifuged, and treated with red blood cell lysis buffer (ACK lysis buffer, Gibco, Billings MT). Afterward, RPMI medium was added, and the cells were filtered again, centrifuged, and resuspended in flow wash buffer (Invitrogen). For T cell preparation, cells were incubated with a cell stimulation cocktail with a Golgi block (eBioscience, San Diego CA) in RPMI with 10% fetal calf serum (FCS) at 37°C with 5% CO2 for 6 hours. To prepare for staining, the cells were first incubated with a fixable viability dye on ice for 30 minutes. Following centrifugation and washing, the cells were treated with an Fc receptor-blocking solution (anti-CD16/CD32) for 20 minutes in ice. After centrifugation and discard of the supernatant, dispersed cells were stained with a master mix containing fluorescent antibodies anti-CD45, anti-CD64, anti-CD11b, anti-CD11c, anti-MHCII, anti-CD103, anti-SiglecF, anti-CD24, and a dump gate containing anti-CD3, anti-Ly6g, anti-B220, and viability dye to remove T cells, neutrophils, B cells, and dead cells, respectively (Supplementary Table S1). For the T cell experiments, cells were stained with the following antibody panel: anti-CD45, anti-CD4, anti-CD8, and anti-CD3 (surface staining); anti-IL4, anti-IL17A, and IFN-γ (cytoplasmic staining) and anti-FoxP3 (nuclear staining). Cytoplasmic and nuclear staining were performed using a Bioscience Intracellular Fixation and Permeabilization Buffer Set and FOXP3/Transcription Factor Staining Buffer set according to the manufacturer’s instructions. For Vβ analysis, we used the Mouse Vβ TCR Screening Panel (BD Pharmingen, San Diego CA), which measures the expression of mouse Vβ 2, 3, 4, 5.1 and 5.2, 6, 7, 8.1 and 8.2, 8.3, 9, 10b, 11, 12, 13, 14, and 17a T-cell. For analysis, we measured the fluorescence intensity of each Vβ antibody on the CD3+CD4 + population and calculated the percentage of cells expressing each Vβ family.

### Statistics

All results are reported as mean ± SD. Differences between two groups were assessed by paired or unpaired *t*-test; for ≥3 groups, differences were assessed by ANOVA followed by *post hoc* Tukey test. *P* values <0.05 were considered statistically significant. Prism (v10.1, GraphPad, San Diego, CA) was used for statistical analyses and graphing.

## Results

### *S. mansoni* exposure increases DC density in the lung parenchyma

**Figure 1:**
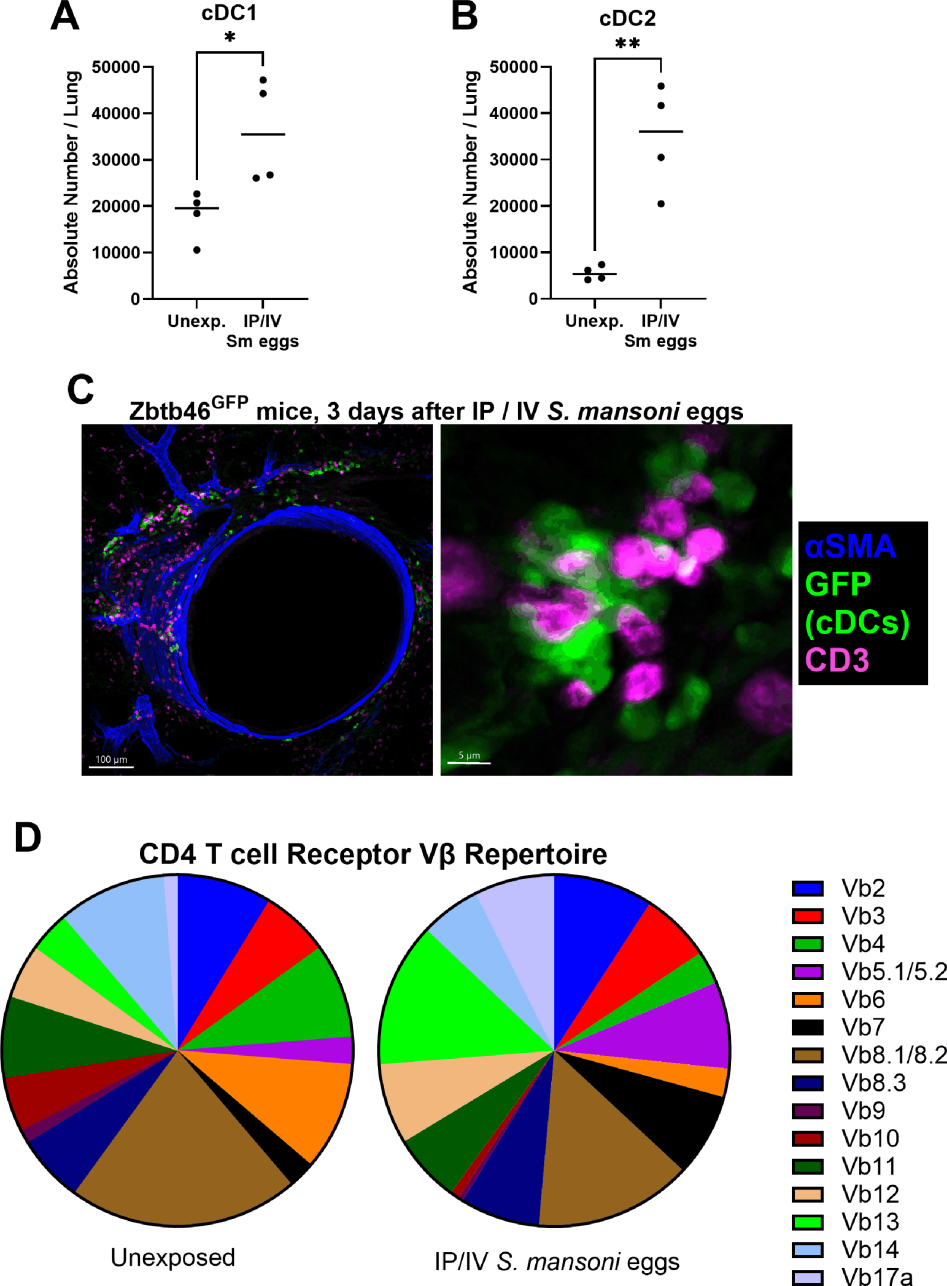
Conventional dendritic cells (cDCs) are increased and in proximity to T cells in mice with *Schistosoma*-induced PH, and the T cell repertoire shifts in CD4 T cells. Total number of (**A**) cDC1s and (**B**) cDC2s in the lungs with *Schistosoma*-induced PH mice (*N* = 4/group; unpaired *t*-test, **P<0.01, ** *P*<0.01. (**C**) Identification of cDCs (both types) with *Zbtb46^GFP^* reporter mice, alpha-smooth muscle actin (αSMA) and CD3 (all T cells). (**D**) Expression pattern of Vβ chains among CD4 T cells in control and *Schistosoma*-PH mice (pooled from *N* = 3 mice per group).

We quantified the number of cDC1 and cDC2 in the lung parenchyma using flow cytometry, using the gating strategy shown in Supplementary Figure S1. We observed in wildtype mice IP sensitized and IV challenged with *S. mansoni* eggs a significant increase in the density of both cDC1s (2.0-fold, *t*-test *P*=0.028) and cDC2s (6.3-fold, *P*=0.002; Figure 1A and B).

Zbtb46 is a transcription factor required for the development of both cDC subtypes. Using *Zbtb46^gfp^* reporter mice, we observed perivascular cDCs, which were often located in close proximity to CD3-expressing T cells consistent with interactions between peptide-MHC II (pMHCII) complexes and T cell receptors (TCRs) (Figure 1C).

### The lung CD4 T cell receptor repertoire shifts in *S. mansoni*-exposed mice

CD4 T cells activated in response to infectious diseases undergo proliferation in those cells with cognate TCRs to pMHCII. We previously observed an increase in T effector memory cells in *S. mansoni*-PH mice [3]. We sought further evidence of CD4 T cell activation by assessing the distribution of the TCR Vβ chain repertoire using flow cytometry, observing a shift in mice sensitized and challenged with *S. mansoni* eggs, consistent with activation and clonal proliferation of CD4 T cells (Figure 1D).

### Deletion of both cDCs does not significantly affect *S. mansoni*-induced PH

**Figure 2:**
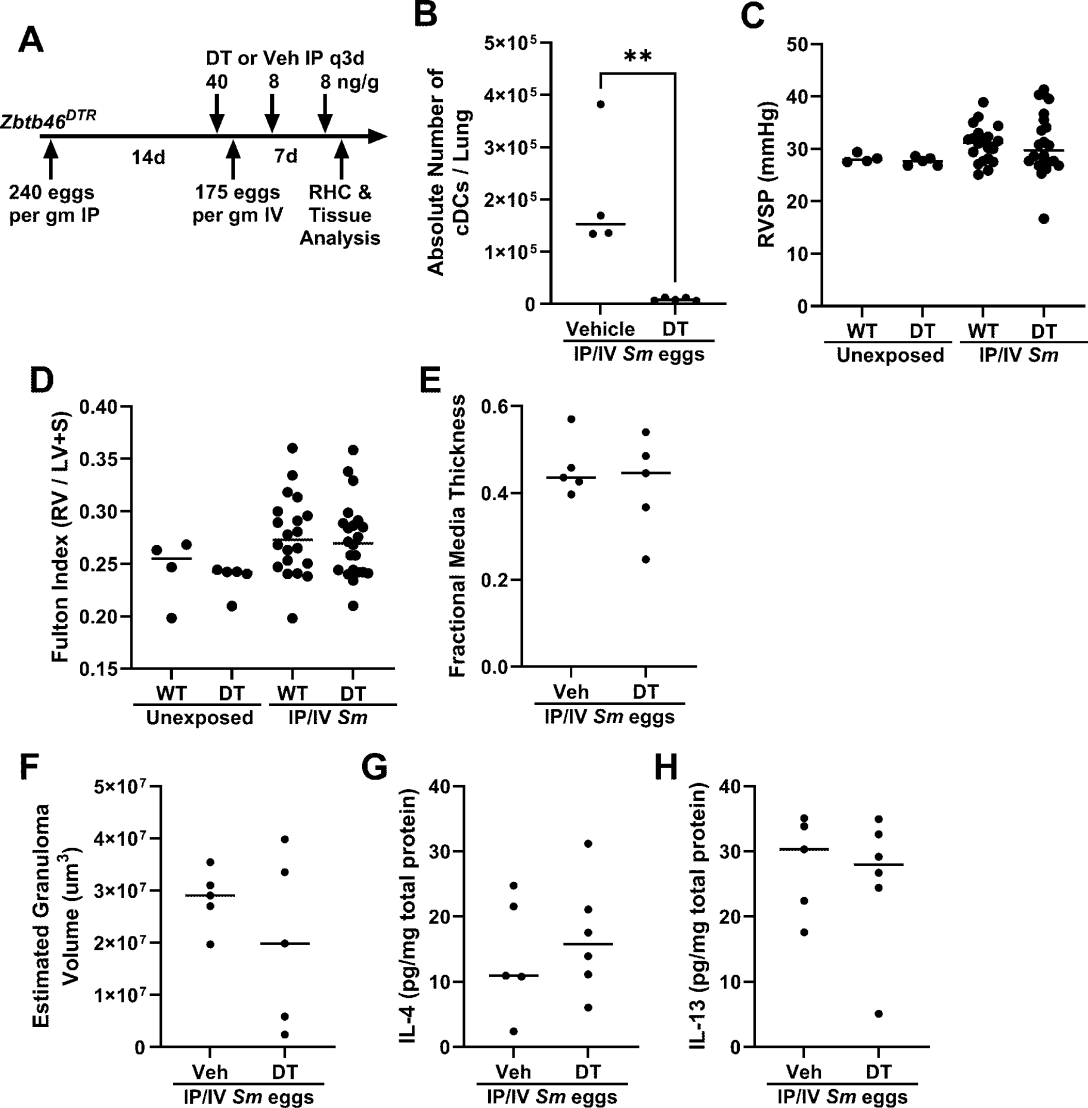
*Zbtb46^DTR^* **mice are not significantly protected** from *Schistosoma* **-induced PH or Type 2 inflammation**. (**A**) Schematic of experimental design. (**B**) Quantification of deletion efficiency of cDCs. (**C**) RVSP, (**D**) Fulton index, (**E**) fractional media thickness, (**F**) estimated peri-egg granuloma volume, and total lung homogenate concentrations of (**G**) IL-4 and (**H**) IL-13. ANOVA and unpaired *t*-test; **P<0.01; all other *P*>0.05.

We used Zbtb46^DTR^ chimeric mice (transplant of Zbtb46^DTR^ bone marrow into lethally irradiated wildtype mice) to delete both cDC subsets when administered DT [19], prior to administering IP *S. mansoni* egg sensitization and IV *S. mansoni* egg challenge (Figure 2A). We observed a significant 96% decrease in the number of cDCs in the lung tissue (Figure 2B). We observed no significant change in the PH phenotype by RVSP, RV hypertrophy, or vascular remodeling (Figure 2C–E). The peri-egg granuloma volumes and concentrations of IL-4 and IL-13 were not significantly suppressed by DT treatment (Figure 2F–H).

### cDC1s are protective in *S. mansoni*-induced PH

**Figure 3:**
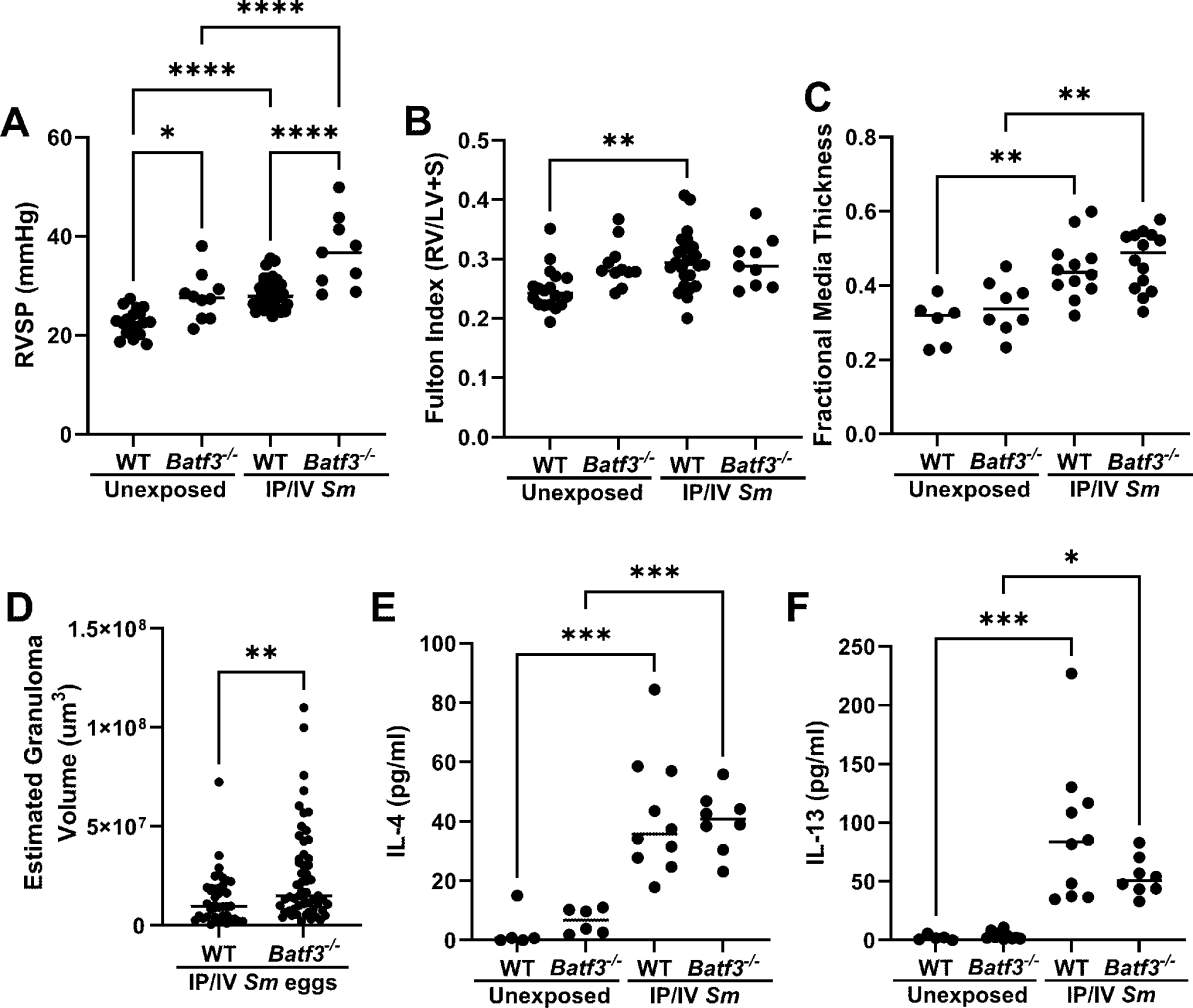
cDC1-deficient Batf3*^-/-^* mice have more severe *Schistosoma mansoni*-induced PH. (**A**) RVSP, (**B**) Fulton index, (**C**) fractional media thickness, (**D**) estimated peri-egg granuloma volume, and total lung homogenate concentrations of (**E**) IL-4 and (**F**) IL-13. (**A-C**): ANOVA with *post hoc* Tukey testing. (**D-F**): unpaired *t*-test. *P* values: *P<0.05; **P<0.01; ***P<0.001; ****P<0.0001.

We used *Batf3^-/-^* mice to interrogate the role of cDC1s. We observed at baseline a modest PH phenotype in these mice with spontaneously higher RVSP (Figure 3A), consistent with a potential role of cDC1s in pulmonary development. Upon *S. mansoni* sensitization and challenge, we observed a much higher RVSP than in wildtype *S. mansoni* sensitized and challenged mice, indicating that cDC1s are protective. The degree of RV hypertrophy and vascular remodeling was unchanged (Figure 3B and C). Consistent with the higher RVSP, the size of peri-vascular granulomas was modestly larger, although the concentrations of IL-4 and IL-13 were unchanged in *S. mansoni*-exposed mice (Figure 3D–F). Of note, the density of cDC2s was not altered in the *Batf3^-/-^* mice (Supplementary Figure S2).

As it was previously reported that *Batf3^-/-^* had more severe *S. mansoni*-induced liver disease but were protected from *S. japonicum*-liver disease, we assessed the PH phenotype of *Batf3^-/-^* mice in *S. japonicum*-induced PH. We observed that cDC1s were largely dispensable, in that the PH severity and degree of Type 2 inflammation were unchanged in *Batf3^-/-^* (except IL-13 concentration was lower) as compared with wildtype mice sensitized and challenged with *S. japonicum* eggs (Supplementary Figure S3).

**Figure 4:**
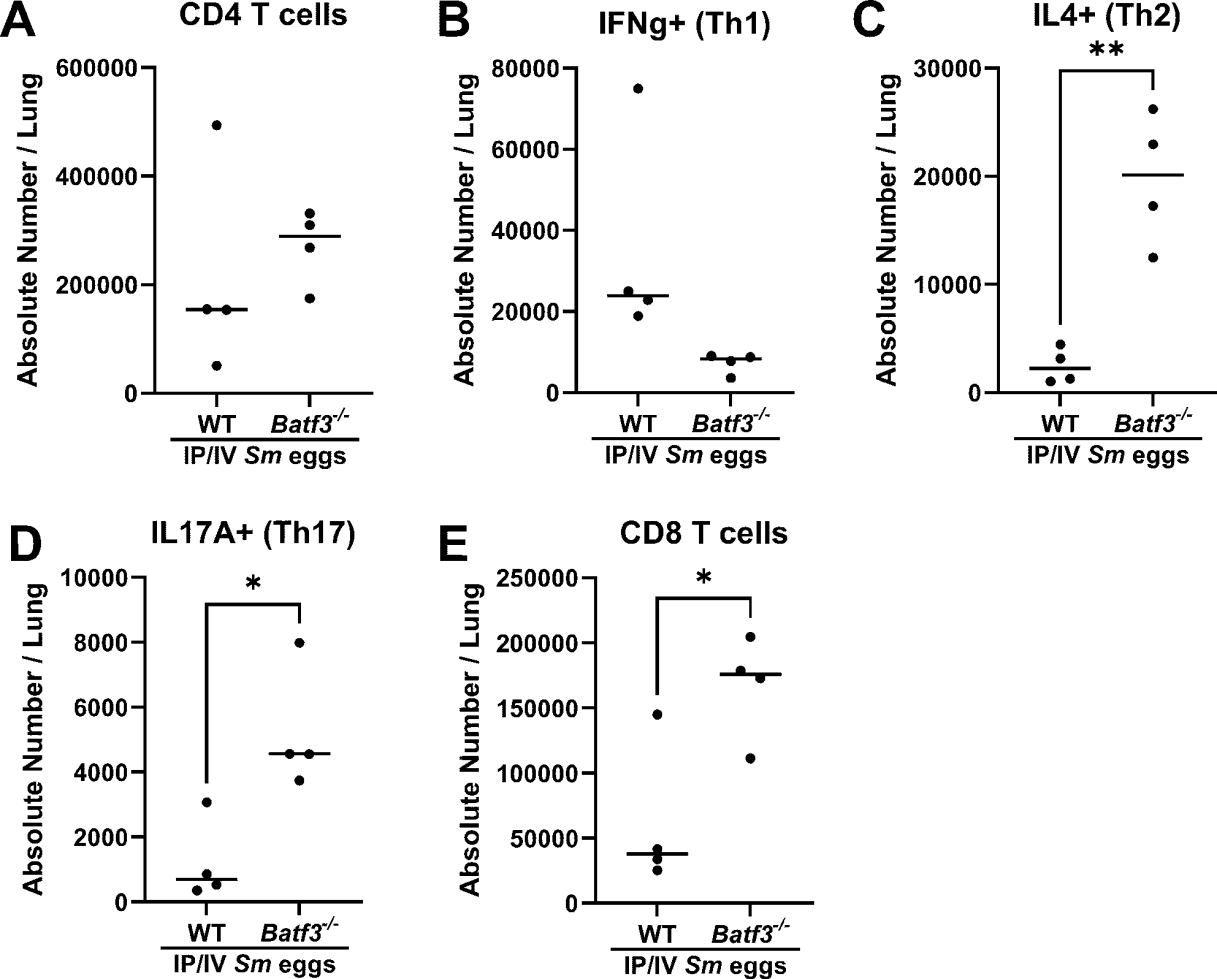
*Batf3^-/-^* **mice** with *Schistosoma* **-PH have a shift in pulmonary CD4 T cell phenotypes**. (**A**) All CD4 T cells, and subsets of (**B**) IFN-g+ (Th1), (**C**) IL-4+ (Th2), (**D**) IL-17A+ (Th17). (**E**) All CD8 T cells. Unpaired *t*-test; *P* values: *P<0.05; **P<0.01.

Considering the potential mechanism by which cDC1s may be protective, we assessed by flow cytometry the T cell phenotype in *Batf3^-/-^* mice sensitized and challenged with *S. mansoni*. We observed an overall unchanged density of CD4 T cells, but a significant increase in Th2 and Th17 CD4 T cells (Figure 4). We also observed a higher number of CD8 T cells.

**Figure 5:**
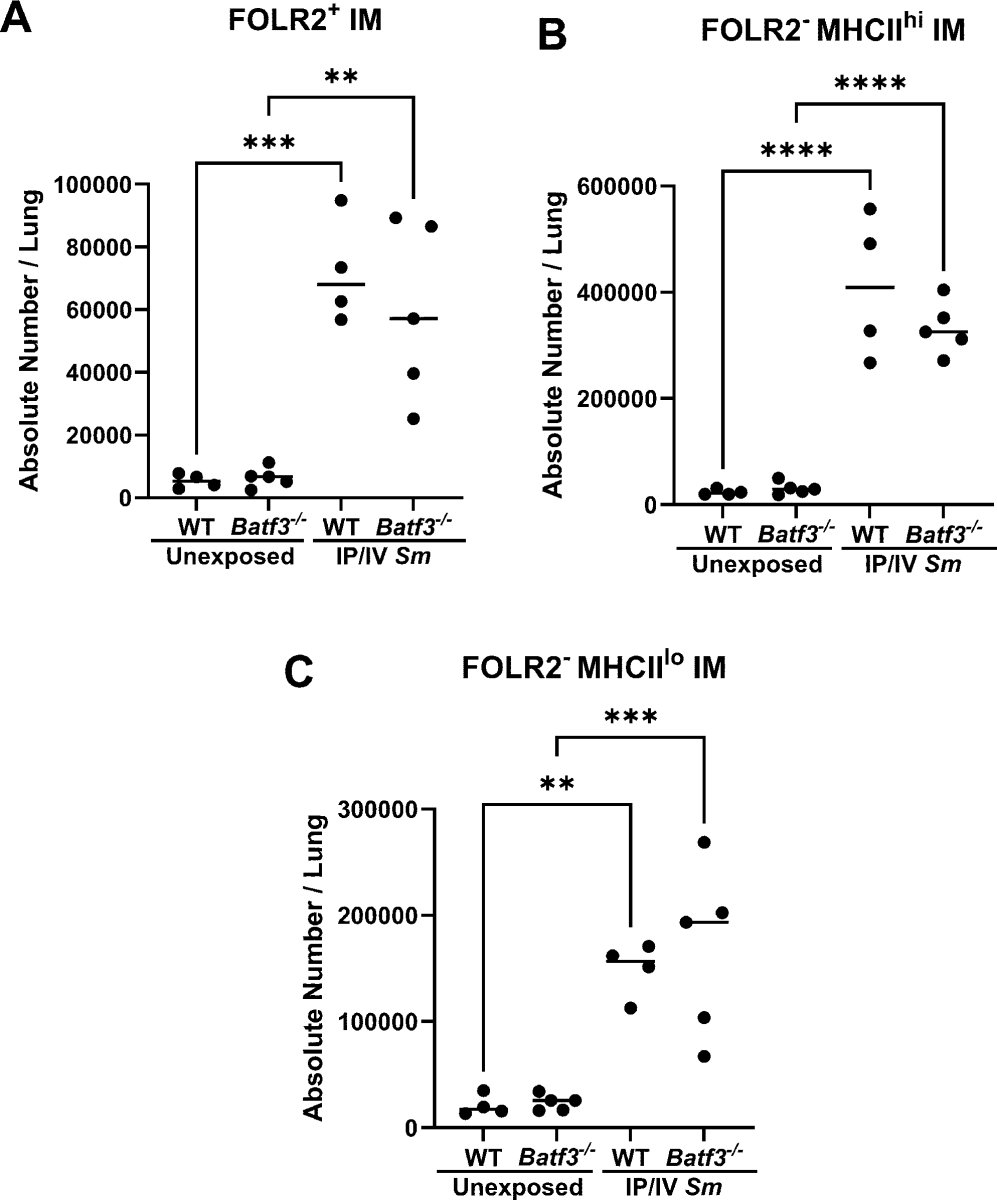
*Batf3^-/-^* **mice** with *Schistosoma* **-PH do not have significantly different pulmonary interstitial macrophage (IM) phenotypes**. (**A**) FOLR2^+^, (**B**) FOLR2^-^MHCII^hi^, and (**C**) FOLR2-MHCII^lo^ IM subpopulations. ANOVA with *post hoc* Tukey test; *P* values: *P<0.05; **P<0.01; ****P<0.0001. See Supplementary Figure S1 for gating strategy.

Having previously observed a requirement for interstitial macrophages (IMs) to promote *Schistosoma*-PH, we assessed the density of the 3 IM subpopulations that have been described [20,21]. We observed a rise in all three IM subpopulations in both WT and *Batf3^-/-^* mice following *Schistosoma* exposure, but no significant differences between the wildtype and *Batf3^-/-^* genotypes (Figure 5).

### cDC2s promote pathology in *S. mansoni*-induced PH

CD301b is a transcription factor specific to cDC2s; *Cd301b^DTR^* mice treated with DT have significant depletion of cDC2s [19]. We first attempted to delete cDC2s starting at the time of IP egg sensitization and continuing through IV egg challenge to the time of heart catheterization (Supplementary Figure S4A). However, we found this prolonged DT treatment induced a paradoxical increase in the density of cDC2s, although not cDC1s (Supplementary Figure S4B), likely due to either a cumulative toxic effect of DT, and/or up-regulation of other pathways that rescued the cDC2 cell population. This increase in cDC2s was associated with a trend toward increased RVSP, and increased vascular remodeling and total lung IL-4 concentration (Supplementary Figure S4D-H), overall making the results difficult to interpret.

**Figure 6:**
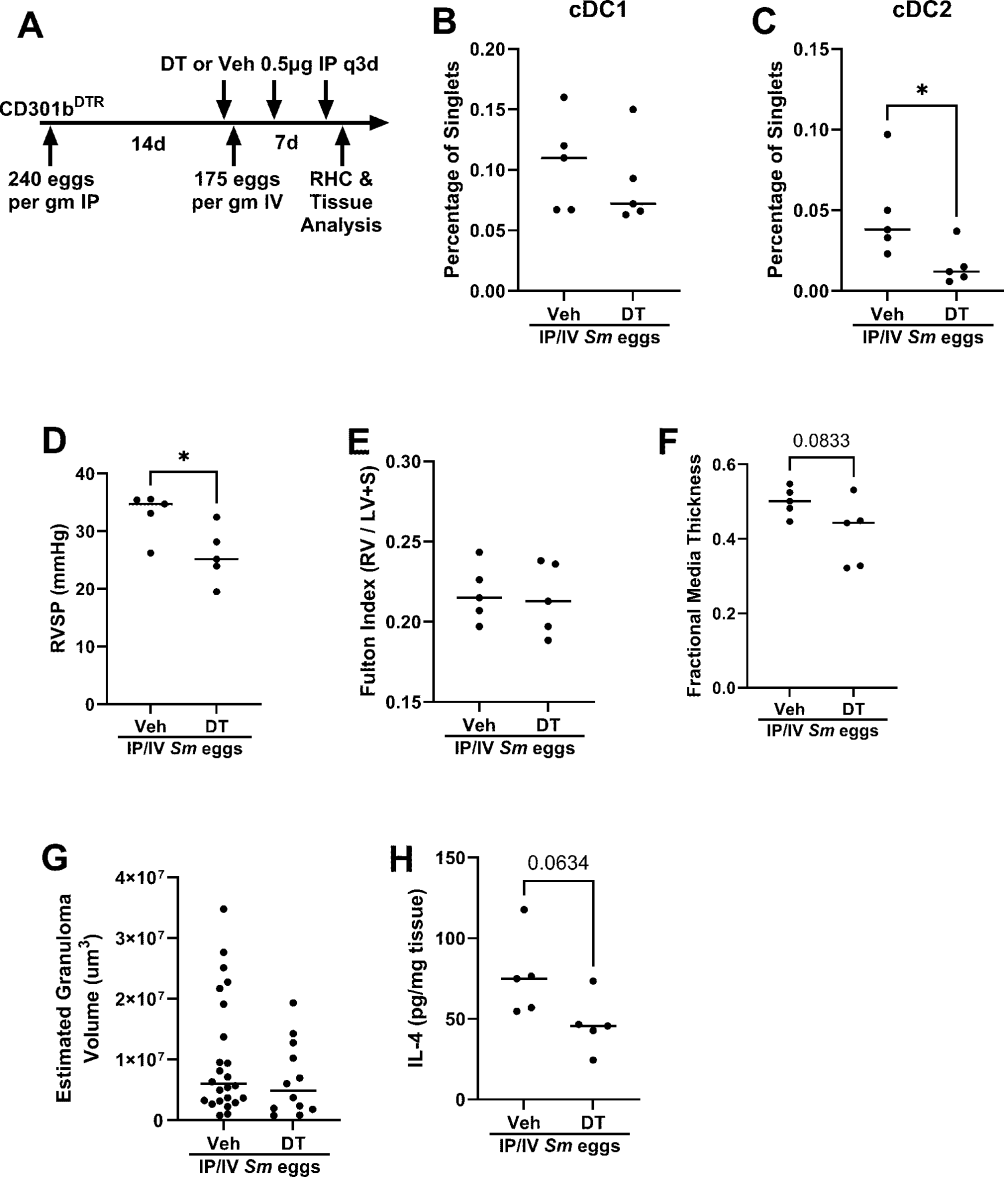
*Cd301b^DTR^* **mice with depleted cDC2s are protected** from *Schistosoma* **-induced PH**. (**A**) Schematic of the experimental design. Total number of (**B**) cDC1s and (**C**) cDC2s in the lung parenchyma. (**D**) RVSP, (**E**) RV hypertrophy by Fulton index, (**F**) fractional media thickness, (**G**) peri-egg granuloma volumes, and (**H**) IL-4 concentration in lung lysates. Unpaired *t*-test; *P* values: *P<0.05.

Instead, we administered DT after IP sensitization and prior to IV eggs challenge (Figure 6A). We observed a heterogeneous effect, with five of eight mice decreasing their cDC2 density by about 65% on average, compared with vehicle-treated mice, without changing the cDC1 density (Figure 6B and C). Mice treated in this manner had decreased PH severity, with a significant reduction in RVSP and a trend (*P*=0.083) toward a reduction in vascular remodeling (Figure 6D–F). Of note, in the three mice that did not decrease their cDC2 density, the average RVSP remained elevated at 30 mmHg. The size of estimated peri-egg granuloma volumes did not decrease (Figure 6G), but the concentration of IL-4 decreased about 40% in the DT-treated mice (*P*=0.063; Figure 6H).

Considering that DT alone might have an effect, we administered DT, or vehicle, to wildtype mice sensitized and challenged in the same manner as the *Cd301b^DTR^* mice treated starting just prior to IV egg challenge. This resulted in no significant impact on the PH phenotype (Supplementary Figure S5A and B)**.** The whole lung IL-4 concentration increased, but granuloma volumes were unchanged (Supplementary Figure S5C and D)**.**

## Discussion

**Figure 7:**
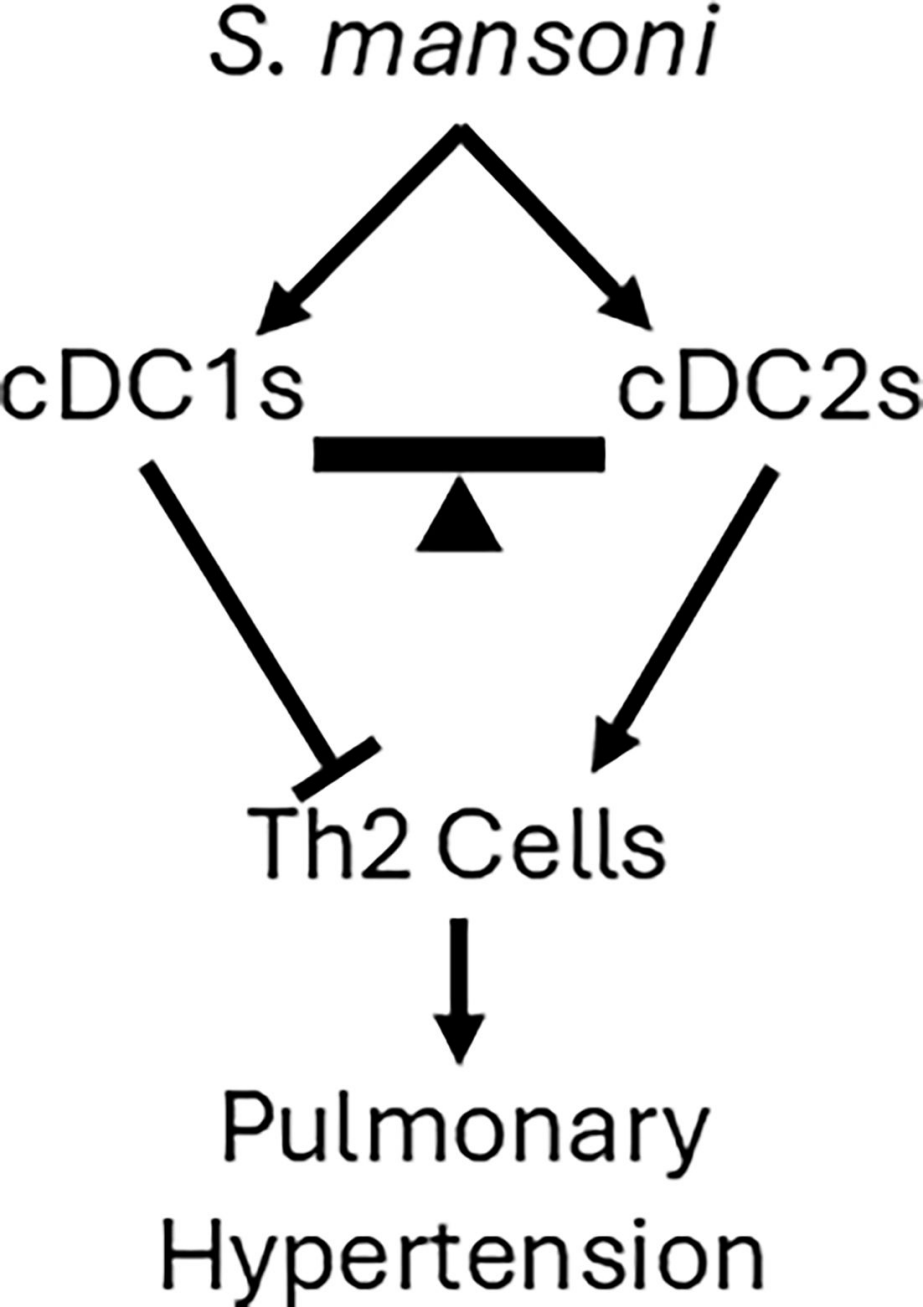
Summary of the model for the dual role of cDCs in *Schistosoma*-induced PH.

In this study, we investigated the role of conventional DCs in *Schistosoma*-PH. We observed a significant increase in the density of both cDC1s and cDC2s in *Schistosoma-exposed* mice, but deletion of both together did not significantly affect the PH phenotype. However, deletion of cDC1s alone caused worse *S. mansoni*-induced PH, associated with more significant Th2 inflammation, whereas depletion of cDC2s alone was protective against *S. mansoni*-induced PH. This suggests a working model in which cDC1s constrain, whereas cDC2s promote Th2-driven *Schistosoma*-induced PH (Figure 7).

PH is an uncommon complication of schistosomiasis, thought to affect about 1% of those with chronic *Schistosoma* infection [22]. However, as PH from any etiology is relatively uncommon, and about 250M worldwide have schistosomiasis, *Schistosoma* is a cause of many cases of PH worldwide. Schistosoma is endemic in 74 countries worldwide, with 90% of cases occurring in sub-Saharan Africa. It has been reported that patients with *Schistosoma*-PH may have a more favorable outcome than those with idiopathic PH [23], but newer data from endemic settings suggest the survival could be comparable [24]. *Schistosoma*-PH can be treated with the same medications used to treat other forms of Group 1 PH [25], but specific disease-modifying therapies are lacking.

Our study indicates important functional differences in these two cDC subsets in *Schistosoma*-PH. These functional differences probably derive from different developmental pathways: cDC1s are Batf3 dependent, whereas cDC2s are IRF4 and KLF4 dependent. These transcriptional factors give rise to different expressed proteins that affect function: cDC1s express CD8a, CLEC9a, and Xcr1; whereas cDC2s express CD301b, CD11b, and SIRPa. Functionally, cDC1s are able to cross-present antigen to CD8 T cells.

In *S. mansoni*-induced PH, cDC1s appear to restrain Th2 immunity, potentially mediated by interactions with CD4 and/or CD8 T cells [4–6,26]. Prior work in *S. mansoni*-liver disease in cDC1-deficient Batf3^-/-^ mice observed a protective role of anti-inflammatory IL-10-expressing CD8 T cells [11]. These data are similar to *Leishmania* infection, in which *Batf3^-/-^* mice had decreased Th1 and increased Th2 inflammation [27,28].

In contrast, cDC2s probably contribute to antigen presentation to primed CD4 T cells in IP egg-sensitized mice. Th2 cells require antigen presentation to become activated. Primed CD4 T cells do not require professional APCs, and many cells can potentially present antigen, including endothelial cells and macrophages [29,30]. This may explain why the effect of cDC2 deletion was only partial. We observed a modest reduction in IL-4, which paralleled the reduction in RVSP after cDC2 deletion; of note, we have previously observed that in *Schistosoma*-exposed mice, IL-4 correlates with RVSP, to a degree even better than IL-13 [1].

The mechanisms by which cDC1s are protective in *S. mansoni*-induced PH but dispensable in *S. japonicum*-induced PH are unclear. Overall, it appears that *S. japonicum* induces more mild PH and Type 2 inflammation severity than *S. mansoni* [13]*, and it may be that the degree of inflammation affects the functional role of cDC1s*.

cDCs have been investigated in other parasitic infections as well, in addition to the leishmaniasis data mentioned above. *Toxoplasma gondii* infection in mice increased the number and activation of both cDC1s and cDC2s [31]. In mice infected with the rodent malarial parasite *Plasmodium berghei,* both cDC1 and cDC2 demonstrated decreased cytokine expression in both subsets but increased phagocytic capacity uniquely in cDC2s after anti-malarial treatment [32]. Mice infected with *Cryptosporidium parvum* have an increase in cDC1s (cDC2s were not investigated), which contribute to clearance of the parasite [33]. In the context of *Nippostrongylus brasiliensis* infection, deletion of Wnt4 in DCs particularly suppressed cDC1s and increased cDC2s, associated with increased IL-5 and more ILC2s, and more effective worm expulsion [34]. Overall, these data suggest the function of cDC1 and cDC2 can be very context-dependent in the setting of parasitic infections.

Prior studies investigating how *Schistosoma* antigens are processed by murine DCs have primarily utilized *ex vivo* systems, principally CD11c^+^MHCII^+^ splenocytes [35–38], which are likely a mixture of both cDC populations. These *ex vivo* studies have questioned if antigen presentation is required, as *Schistosoma* antigens functionally condition DCs for Th2 priming, but do not clearly cause DC activation or maturation themselves [36]. The observed phenotype may be mediated by RNase functions of internalized egg proteins, which modify DCs by degrading host rRNAs and mRNAs [35]. Mice treated with the *S. mansoni* protein omega-1 had cDC2s with decreased migratory capacity that was dependent on the RNAse function of omega-1 [39].

The contribution of cDC2s to PH pathogenesis is also shared with experimental hypoxia-induced PH, in which *Cd301b^DTR^* mice were similarly protected from a rise in RVSP [40]. Notably, hypoxia-induced PH is associated with a Th17 phenotype [41], different than Th2 as is critical in *Schistosoma*-PH. Further, cDC1s appear to be dispensable in hypoxia-induced PH, as *Batf3^-/-^* mice did not have a different PH phenotype than wildtype mice after hypoxia exposure [40]. This is similar to our observation that cDC1s are dispensable for *S. japonicum*-induced PH. In some, these data suggest the role of DCs in PH pathogenesis is context-dependent, dependent on the specific trigger.

Important limitations include less than complete deletion of the cDC populations using the transgenic approaches herein. Part of this may be due to the effect of repeated doses of DT over the experimental duration, which appears to induce a degree of Th2 immunity on its own and may result in DC up-regulation by other mechanisms. We have also not interrogated the potential roles of plasmacytoid or monocyte-derived DCs. Most of the experiments were performed at Denver elevation, including the control groups, but there is a possibility that the mild hypoxia (equivalent to about 18% FiO2 vs. 21% at sea level) could confound the observed results—for example, mild baseline PH could mask the effect of DC deletion. We also did not test whether there was a baseline phenotype in the CD301b-DTR mice, although we did exclude a significant effect of DT treatment alone in wildtype mice. We also used an open chest technique to assess RVSP, which requires deeper sedation.

The mechanism by which PH severity worsens in the Batf3^-/-^ mice is still uncertain, such as by CD8-dependent mechanisms seen in liver pathology [11]. It is also unclear why the phenotype of cDC1 deficiency differs between *S. japonicum* and *S. mansoni*: it could be related to the degree of inflammation in *S. japonicum* (which we found is generally less [13]) or specific parasite-degree antigens. It would be interesting to determine potentially alternative APCs in the context of cDC2 deficiency that are able to compensate at least in part. Having observed that cDC2s contribute to both hypoxia-induced [40] and now *Schistosoma*-induced PH, the relevance of these cells in other PH models should be tested, such as following monocrotaline or SU5416-hypoxia.

In conclusion, we observe cDC1s are protective in *S. mansoni*-induced PH, but dispensable in *S. japonicum*-induced PH. In contrast, cDC2s contribute to the Th2 inflammation and PH that follows IV-delivered *S. mansoni* eggs in sensitized mice.

**Clinical Perspectives**

- Schistosomiasis is a major but not well-understood cause of pulmonary hypertension (PH) worldwide. CD4 T cells are required for *Schistosoma*-induced PH in mice. This study was undertaken to investigate the role of conventional dendritic cells (cDCs), which activate T cells.
- We found that both subsets of cDCs were increased in *Schistosoma mansoni*-exposed mice, but deleting both at the same time did not affect the PH severity. However, deletion of just Type 1 cDCs caused increased Th2 inflammation and PH, whereas deletion of just Type 2 cDCs was protective against PH.
- These results shed important insights on the inflammatory pathogenesis of schistosomiasis-associated PH. Blocking the Th2 inflammatory cascade may be beneficial in preventing or treating this condition.

## Supplementary material

Online supplementary material 1

## Data Availability

Requests for resources and reagents can be addressed directly to the corresponding authors. All data generated or analyzed during this study that are not included in this published article and its supplementary information are available from the corresponding author on reasonable request.

## Abbreviations

DCs: dendritic cells
PH: pulmonary hypertension
SchPH: *Schistosoma*-induced PH
cDCs: conventional dendritic cells
pDCs: plasmacytoid DCs
