# Supplementary material for: Role of conventional dendritic cells in schistosomiasis-induced pulmonary hypertension
Source: Clin Sci (Lond). 2025 Oct 14;139(20):CS20256896. doi: 10.1042/CS20256896 (PMC12687420; doi:10.1042/CS20256896)
Supplement: Online supplementary material 1 [file cs-139-20-CS20256896-s001.docx]

**SUPPLEMENTAL TABLES AND FIGURES**

Role of Conventional Dendritic Cells in Schistosomiasis-Induced Pulmonary Hypertension

*Mickael et al*

**Supplemental Table 1. Antibodies used for murine flow cytometry studies.**

| **Antibody Specificity** | **Fluorochrome** | **Clone** | **Final Conc.** | **Manufacturer** |
| --- | --- | --- | --- | --- |
| Anti-CD3 | APC/C7 | 17A2 | 1/100 | Tonbobiosciences |
| Anti-CD3 | eFluor™450 | 17A2 | 1/100 | Invitrogen |
| Anti-CD4 | BV510 | RM4-5 | 1/100 | BioLegend |
| Anti-IL4 | Alexa™Fluor 488 | 11B11 | 1/100 | BDBiosciences |
| Anti-IL17A | APC | eBio1787 | 1/100 | Invitrogen |
| Anti-IFNγ | PE | XMG1.2 | 1/100 | Invitrogen |
| Anti-FoxP3 | PE | MF23 | 1/100 | BD Pharmingen™ |
| Anti- IV CD45 | PerCp | 104 | 1/100 | BioLegend |
| Anti-CD45 | BV650 | 104 | 1/100 | BioLegend |
| Anti-IA/IB | APC/Cy7 | M5/114.15.2 | 1/100 | BioLegend |
| Anti-B220 | eFluor™450 | RA-3682 | 1/100 | Invitrogen |
| Anti-Ly6G | eFluor™450 | 1AB-Ly6g | 1/100 | Invitrogen |
| Anti-Siglec-F | PE-CF594 | E50-2440 | 1/100 | BD Horizon |
| Anti-CD11b | BV510 | M1/70 | 1/100 | BD Horizon |
| Anti-CD11c | PE/Cy7 | N418 | 1/100 | Invitrogen |
| Anti-CD64 | APC | X54-5/7.1 | 1/100 | BioLegend |
| Anti-CD103 | BV605 | 2E7 | 1/100 | BioLegend |
| Anti-CD301b | PE | URA-1 | 1/100 | BioLegend |
| Anti-CD24 | FITC | M1/69 | 1/100 | BioLegend |
| Anti-CCR2 | AF700 | SA203G11 | 1/50 | R&D Systems |
| Anti-CCR2 | PE/Cy5 | SA203G11 | 1/50 | BioLegend |
| anti-mouse Vβ 2 TCR | FITC | B20 .6 | 20ul/ 10^6^ cells | BDBiosciences |
| anti-mouse Vβ 3 TCR | FITC | KJ25 | 20ul/ 10^6^ cells | BDBiosciences |
| anti-mouse Vβ 4 TCR | FITC | KT4 | 20ul/ 10^6^ cells | BDBiosciences |
| anti-mouse Vβ 5.1, 5.2 TCR | FITC | MR9-4 | 20ul/ 10^6^ cells | BDBiosciences |
| anti-mouse Vβ 6 TCR | FITC | RR4-7 | 20ul/ 10^6^ cells | BDBiosciences |
| anti-mouse Vβ 7 TCR | FITC | TR310 | 20ul/ 10^6^ cells | BDBiosciences |
| anti-mouse Vβ 8.1, 8.2 TCR | FITC | MR5-2 | 20ul/ 10^6^ cells | BDBiosciences |
| anti-mouse Vβ 8.3 TCR | FITC | 1B3 .3 | 20ul/ 10^6^ cells | BDBiosciences |
| anti-mouse Vβ 9 TCR | FITC | MR10-2 | 20ul/ 10^6^ cells | BDBiosciences |
| anti-mouse Vβ 10b TCR | FITC | B21 .5 | 20ul/ 10^6^ cells | BDBiosciences |
| anti-mouse Vβ 11 TCR | FITC | RR3-15 | 20ul/ 10^6^ cells | BDBiosciences |
| anti-mouse Vβ 12 TCR | FITC | MR11-1 | 20ul/ 10^6^ cells | BDBiosciences |
| anti-mouse Vβ 13 TCR | FITC | MR12-3 | 20ul/ 10^6^ cells | BDBiosciences |
| anti-mouse Vβ 14 TCR | FITC | 14-2 | 20ul/ 10^6^ cells | BDBiosciences |
| anti-mouse Vβ 17a TCR | FITC | KJ23 | 20ul/ 10^6^ cells | BDBiosciences |

**
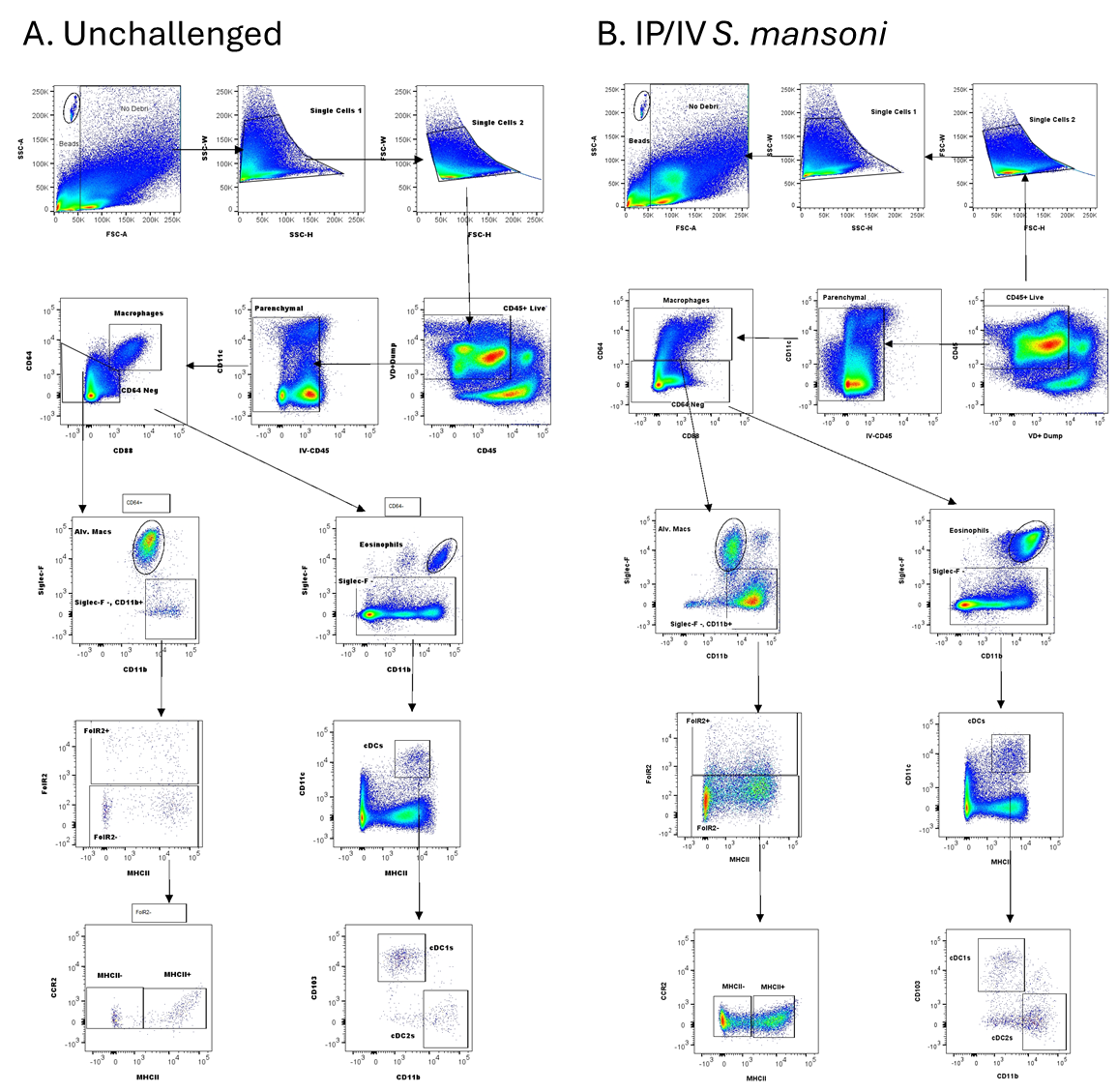
**

**Supplemental Figure 1. Flow cytometry gating strategy to quantify cDC1s and cDC2s, and interstitial macrophages**. Representative (A) unchallenged and (B) *S. mansoni* IP egg sensitized and IV egg challenged wildtype mice.


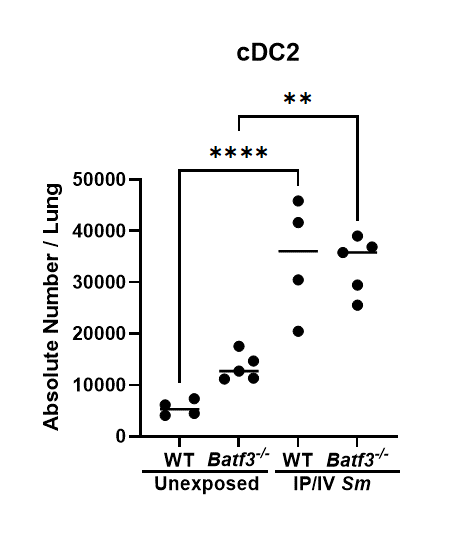


**Supplemental Figure 2. The density of cDC2s is not significantly altered in *Batf3^-/-^* mice.** Total number of cDC2s in wildtype (WT) and cDC1-deficient *Batf3^-/-^* mice. Note that the wildtype data is the same as in Figure 1B. N=4-5/group; ANOVA with post-hoc Tukey test shown; **: *P*<0.01; ***: *P*<0.001; ****: *P*<0.0001.


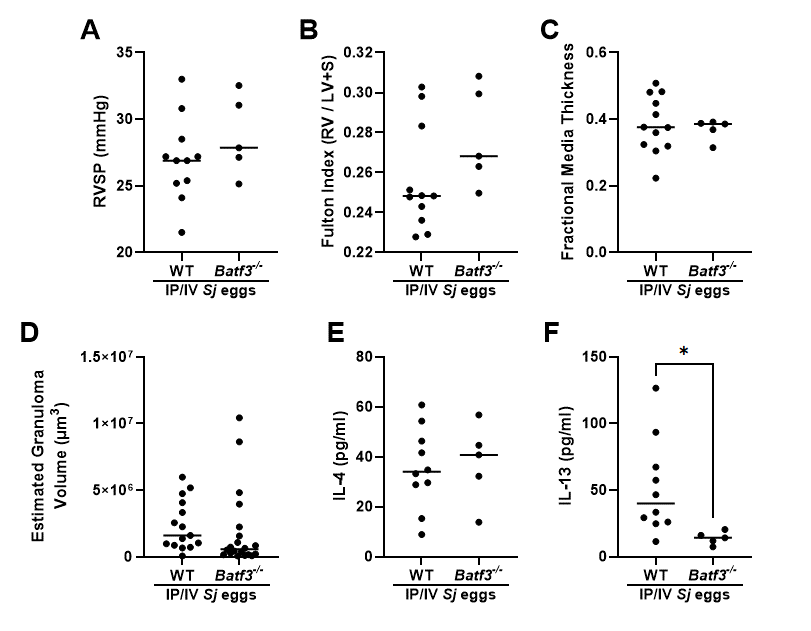


**Supplemental Figure 3. cDC1s are largely dispensable in *Schistosoma japonicum*-induced PH.** (A) RVSP, (B) Fulton index, (C) fractional media thickness, (D) estimated peri-egg granuloma volume, and total lung homogenate concentrations of (E) IL-4 and (F) IL-13 in wildtype (WT) and *Batf3^-/-^* (lacking cDC1s) mice. Note that the wildtype *S. japonicum* data is from Kassa et al ^13^. Unpaired t-test; P values: * P<0.05.


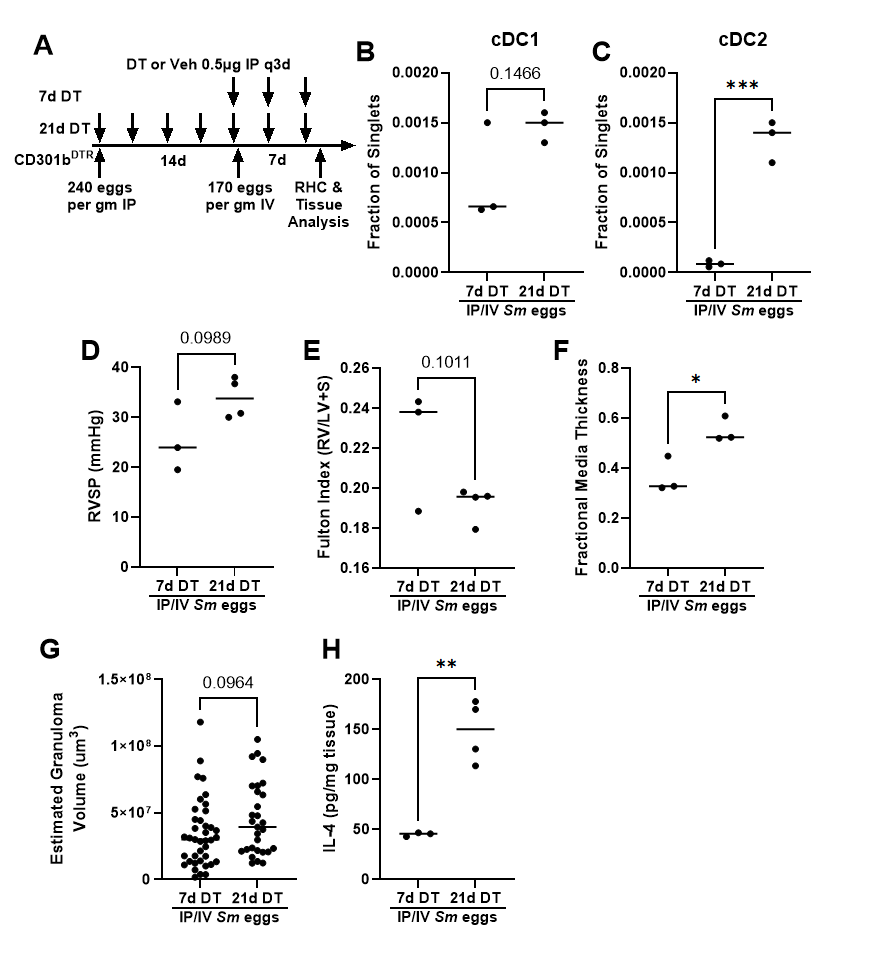


**Supplemental Figure 4. Twenty-one days of diphtheria toxin (DT) treatment in *Cd301b^DTR^* mice does not significantly alter the PH phenotype, but paradoxically increases lung cDC2 density, pulmonary media thickness, and IL-4 expression.** (A) Experimental design schematic. Total number of (B) cDC1s and (C) cDC2s in the lung parenchyma. (D) RVSP, (E) Fulton index, (F) fractional media thickness, (G) estimated peri-egg granuloma volume, and (H) total lung homogenate concentration of IL-4. Unpaired t-test; P values: * P<0.05, ** P<0.01, *** P<0.001.


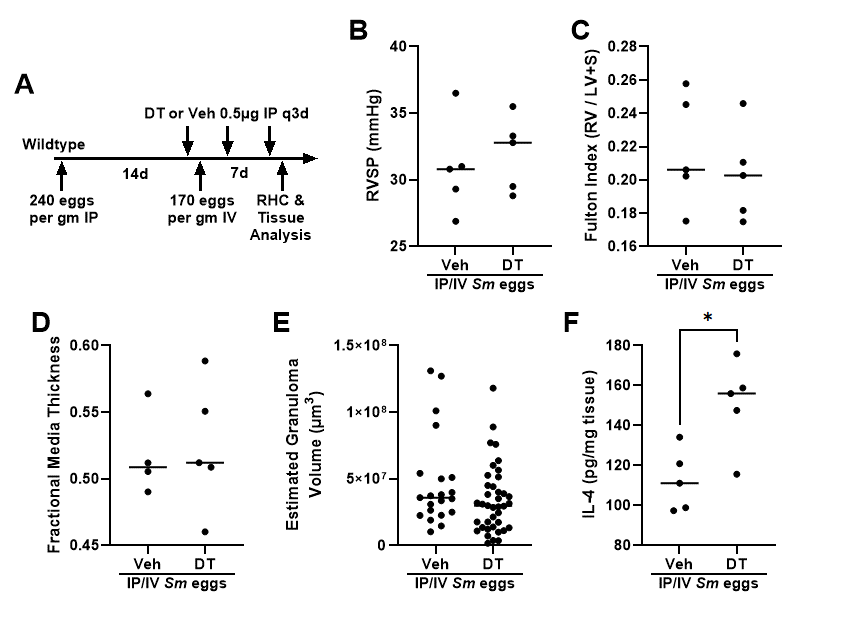


**Supplemental Figure 5. Diphtheria toxin (DT) treatment of wildtype mice does not significantly alter the *Schistosoma*-induced PH phenotype, but increases lung IL-4 expression.** (A) Schematic of experimental design. (B) RVSP, (C) Fulton index, (D) fractional media thickness, (E) estimated peri-egg granuloma volume, and (F) total lung homogenate concentration of IL-4. Unpaired t-test; P values: * P<0.05.
